# Changes in heart rate variability with respect to exercise intensity and time during treadmill running

**DOI:** 10.1186/s12938-018-0561-x

**Published:** 2018-09-24

**Authors:** Kenneth J. Hunt, Jittima Saengsuwan

**Affiliations:** 1 Division of Mechanical Engineering, Department of Engineering and Information Technology, Institute for Rehabilitation and Performance Technology, Bern University of Applied Sciences, 3400 Burgdorf, Switzerland; 2 Department of Rehabilitation Medicine, Srinagarind Hospital and Faculty of Medicine, Khon Kaen University, Khon Kaen, Thailand; 3 Exercise and Sport Sciences Development and Research Group, Khon Kaen University, Khon Kaen, Thailand

**Keywords:** Heart rate control, Heart rate variability, Spectral analysis, Treadmills

## Abstract

**Background:**

Heart rate variability (HRV) arises from the complex interplay of sympathetic and parasympathetic autonomic regulation of heart rate. Ultra-low frequency (ULF) and very-low frequency (VLF) components of HRV play a crucial role in automatic HR controllers, but these frequency bands have hitherto largely been neglected in HRV studies. The aim of this work was to investigate changes in ULF and VLF heart rate variability with respect to exercise intensity and time during treadmill running.

**Methods:**

RR intervals were determined by ECG in 21 healthy male participants at rest, and during moderate and vigorous-intensity treadmill running; each of these three tests had a duration of 45 min. Time dependence of HRV was investigated for moderate and vigorous running intensities by dividing the constant-speed stages into three consecutive windows of equal duration ($\sim$ 14 min), denoted $w_1$, $w_2$ and $w_3$. ULF and VLF power were computed using Lomb-Scargle power spectral density estimates.

**Results:**

For both the ULF and VLF frequency bands, mean power was significantly different between the resting, moderate and vigorous intensity levels (overall $p < 0.001$): mean power was lower for moderate vs. rest ($p < 0.001$), for vigorous vs. rest ($p < 0.001$), and for vigorous vs. moderate ($p < 0.001$). For both ULF and VLF and moderate intensity, mean power was significantly different between the three time windows (overall $p < 0.001$ for ULF, overall $p = 0.041$ for VLF): for ULF, mean power was lower for $w_2$ vs. $w_1$ ($p = 0.031$) and for $w_3$ vs. $w_1$ ($p = 0.001$); for VLF, mean power was lower for $w_3$ vs. $w_1$ ($p = 0.007$). For ULF and vigorous intensity, there was no significant difference in mean power between the three time windows (overall $p = 0.12$). For VLF and vigorous intensity, mean power was significantly different between $w_1$, $w_2$ and $w_3$ (overall $p < 0.001$): mean power was lower for $w_2$ vs. $w_1$ ($p = 0.001$) and for $w_3$ vs. $w_1$ ($p < 0.001$).

**Conclusions:**

The degree of HRV in terms of ULF and VLF power was found to decrease with increasing intensity of exercise. HRV was also observed to decrease over time, but it remains to clarify whether these changes are due to time itself or to increases in HR related to cardiovascular drift. For feedback control applications, attention should be focused on meeting performance targets at low intensity and during the early stages of exercise.

## Background

Heart rate variability (HRV) is ordinarily characterised by beat-to-beat variations in the time between peaks in the QRS complex of the ECG wave, i.e. by variations in the RR interval [1]. A formal set of signal analysis standards for measurement, interpretation and clinical application of HRV has been established [2, 3]; these standards comprise time and frequency-domain methods and combinations thereof.

In the frequency domain, HRV analysis has classically been described for four distinct bands [2, 3]:

1. Ultra-low frequency (ULF), where $f < 0.003$ Hz;
2. Very-low frequency (VLF) with $0.003 \le f < 0.04$ Hz;
3. Low frequency (LF), $0.04 \le f < 0.15$ Hz; and
4. High frequency (HF), $0.15 \le f \le 0.4$ Hz.

Since the frequency $f = 0.003$ Hz at the border between the ULF and VLF bands corresponds to a time period of 333 s, short-term recordings of duration < 5 min are restricted to analysis of VLF, LF and HF characteristics, while the ULF band requires longer-term recording [3]; clinically, portable ECG monitors are employed which typically record for up to 24 h (Holter monitor). It is also noted that HR signals may contain power at frequencies above 0.4 Hz, the upper bound of the HF range: consider a notional HR of 180 beats/min, which is 3 beats/s or $f = 3$ Hz.

It has been widely believed that HF power primarily reflects parasympathetic cardiac drive, that LF power has a predominantly sympathetic component [4], and that the LF/HF ratio can thus be used as a measure of sympatho-vagal balance [5, 6], i.e. the relative contributions of sympathetic and parasympathetic activity. However, this delineation of the different compartments of autonomic nervous system activity and the supposed correspondence with HRV power in the different frequency bands has recently been challenged [7, 8]; it now seems that HRV is the result of more complex sympathetic-parasympathetic interactions that are not yet fully understood [9]. It has furthermore been proposed that ULF and VLF power might be predictors of cardiac health [10], but it has also been pointed out that understanding of the mechanisms involved is presently limited [4].

Although it remains to fully elucidate the complex neural mechanisms of HRV and the associated implications for health, it is clear that HRV is an important phenomenon to be considered in the design of engineering systems employed in support of prescription and implementation of exercise training programmes: contemporary recommendations for exercise duration and intensity use HR for delineation of training regimes [11, 12].

In this regard, the focus in the present work is on the frequency-domain characteristics of HRV during treadmill running; concomitantly, it is intended to apply the knowledge gained to the design and analysis of feedback systems for automatic control of HR during treadmill exercise. It has previously been noted that the principal challenge in the design of controllers for HR is to ensure that the feedback system maintains acceptable performance in the face of disturbances to HR caused by physiological HR variability [13]. To this end, attention is focused here on the ULF and VLF bands: this is because, firstly, HR controllers are usually designed with low-pass characteristics and with a crossover region lying within the VLF band (typically, closed-loop bandwidths are around 0.01 Hz, [13]); secondly, disturbances in the ULF and VLF bands caused by HRV can excite the control signal, i.e. the treadmill speed command, to a degree that would be perceptible to, and possibly unacceptable to, the runner.

Previous investigations of feedback controllers for HR have noted that HRV appears to decrease over time during moderate-to-vigorous intensity exercise of duration 45 min, albeit these observations were obtained indirectly using time-domain measures of closed-loop performance rather than from direct analysis of RR intervals: there were substantial and significant decreases in root-mean-square HR tracking error and in average control signal power [13, 14].

A recent review of HRV responses during exercise concluded twofold: that the primary effect is a pronounced reduction in HRV with increasing exercise intensity, up to a moderate intensity corresponding approximately to the first ventilatory threshold; and, secondarily, that HRV decreases over time, but only during low-to-moderate intensity exercise and when accompanied by cardiovascular drift [15]. However, in terms of frequency-domain analysis, the studies included in the review reported only LF, HF and total power components, presumably due to the relatively short duration of exercise bouts that were investigated. It therefore remains an open question as to how ULF and VLF components of HRV are affected by intensity and duration of exercise.

The aim of the present work was to directly investigate changes in ULF and VLF heart rate variability with respect to exercise intensity and time during treadmill running. For this purpose, a recording duration of 45 min was chosen which is within the recommended range for development and maintenance of cardiorespiratory fitness and which is sufficiently long to capture ULF components, and exercise intensities were studied in accordance with levels used in training-intensity prescription (recommended duration is on the range 20–60 min, and recommended intensity is from ‘moderate’ to ‘vigorous,’ based on heart-rate reserve [11, 12]).

## Methods

### Participants

Twenty-one males on the range 24–51 years participated. Inclusion criteria applied during the selection of this cohort were: male, age between 18 and 60 years, able-bodied and physically healthy. Exclusion criteria were known cardiovascular, pulmonary or musculoskeletal problems that might have interfered with or contraindicated moderate to vigorous intensity treadmill exercise.

### Procedures

For each participant, three ECG measurements were made: 45 min of rest while supine; 45 min of running on a treadmill at moderate intensity; and 45 min running at vigorous intensity. These are referred to in the sequel as test conditions ‘r’, ‘m’ and ‘v’, respectively.

Exercise intensity levels were defined using heart rate reserve (HRR) according to formal guidelines [11, 12]: HRR is the difference between an individual’s maximum and resting heart rates, $\text {HRR} = \text {HR}_\text {max} - \text {HR}_\text {rest}$: moderate intensity is 40–59% of HRR; vigorous intensity is 60–89% of HRR. Maximal heart rate was taken as the age-related prediction given by $\text {HR}_\text {max}$ = 220-age [16].

The resting measurement was carried out on the same day as, but prior to, the first treadmill test and was also used to determine resting HR: this was taken as the mean HR during the 6th min of the resting measurement. The intensity for each participant’s first treadmill test (i.e. m or v) was randomly selected. The second treadmill test was conducted on a separate day at the other intensity.

Each treadmill test was conducted as follows:

1. 10-min warm up running on the treadmill while speed was manually adjusted to find the lower end of the selected intensity range (i.e. 40% of HRR for m, where $\text {HR} = 0.4 \cdot \text {HRR} + \text {HR}_\text {rest}$; or 60% of HRR for v, where $\text {HR} = 0.6 \cdot \text {HRR} + \text {HR}_\text {rest}$); in this stage, HR was monitored using a chest belt.
2. 10-min of rest and fitting of the ECG electrodes.
3. 5-min of recorded rest while standing quietly on the treadmill.
4. 45-min of constant-speed running at the speed determined above for intensity m or v.
5. Up to 10-min cool down at a comfortable walking pace of 3.5 km/h.

Participants were instructed to avoid strenuous exercise, alcohol consumption and smoking in the 24 h preceding each test, to refrain from caffeine consumption in the preceding 12 h, and from partaking of a heavy meal in the 4 h before testing.

From the total of 21 participants, some data sets could not be included in the data analysis due to ECG measurement problems: complete data records were obtained for all three conditions, r, m and v, from 15 participants (thus, $n = 15$ for the analysis of intensity dependence); for condition m from 18 participants ($n = 18$ for analysis of time dependence at intensity m); and for condition v from 15 participants ($n = 15$ for analysis of time dependence at intensity v).

### Measurement instruments

A computer-controlled treadmill was employed (model Venus, h/p/cosmos Sports and Medical GmbH, Nussdorf-Traunstein, Germany). During the warm up stage of each test, HR was monitored using a chest belt (T34, Polar Electro Oy, Kempele, Finland). For ECG measurements while running, the treadmill was controlled directly from the ECG software (below) according to a pre-specified speed profile as described above.

RR intervals were obtained using a wireless ECG recording system with up to 12 leads (custo cardio 100 BT ECG system and the associated custo diagnostic professional software, version 4.3; custo med GmbH, Ottobrunn, Germany). In the present study, a 7-lead subset of the full ECG configuration was employed to allow derivation of RR intervals and heart rate. This involved the application of five electrodes, placed according to the manufacturer’s guidelines: right arm and left arm (RA, LA, positioned just below the collarbones); right leg and left leg (RL, LL, positioned just below the rib cage on each side); and one chest electrode (V$_1$, placed at the 4th intercostal space at the right sternal border).

ECG data were recorded at a sample frequency of 1 kHz. Following each measurement, raw RR intervals were exported with a resolution of 1 ms.

### Outcomes and data processing

From the RR time series, power in the ULF and VLF frequency bands was computed using the Lomb-Scargle least-squares spectral analysis method for spectral density estimation. This method was chosen as it is specifically designed and optimised for non-uniformly-spaced data sets such as RR time series.

Prior to spectral analysis, data sets were processed for artefact detection and replacement, then trend removal, and finally low-pass filtering. Artefacts mainly occur due to the running motion and the effect of this on the ECG electrodes. Artefact detection and replacement was performed using an impulse rejection filter which was proposed for spectral analysis of biomedical signals in general [17], and which was previously applied specifically for the preprocessing of RR time series [18]. In this method, artefact detection is based on a Gaussian test statistic and a user-defined threshold (this was set to 5 in the present study). Artefact replacement uses a median filter with a specified window length (set to the value 10 here). For trend removal, a 3rd-order polynomial fit was estimated and subtracted from the data. Low-pass filtering was performed using a 12th-order Butterworth filter with cutoff frequency 0.4 Hz.

All of the above data preprocessing and spectral analysis was carried out using a custom-designed software tool for HRV analysis implemented in Matlab (The Mathworks, Inc., Natick, USA).

For investigation of the dependence of HRV on intensity (r, m and v), power values were calculated using the 45-min resting measurements and the steady-state portions of the constant-speed-running phases of duration 45 min of the tests at moderate and vigorous intensities.

The dependence of HRV on time was investigated for moderate and vigorous running intensities by dividing the constant-speed stages into three consecutive windows of equal duration ($\sim$ 14 min), denoted as windows $w_1$, $w_2$ and $w_3$.

### Statistical analysis

One-way repeated-measures analysis of variance (ANOVA) was applied to investigate possible differences in mean ULF and VLF power under the different test conditions described above, i.e. differences in HRV with respect to intensity and time. Normality of the data sets was checked using a Kolmogorov-Smirnov test with Lilliefors correction; sphericity was checked using Mauchly’s test.

The significance level for all tests was set to 5% ($\alpha = 0.05$). Whenever the ANOVA indicated the existence of a significant difference (i.e. overall $p < 0.05$), post-hoc pairwise comparisons were carried out using Bonferroni correction.

Prior to statistical analysis, all data sets were log-transformed ($\log _{10}$) to preserve normality: since the average power of a signal *x*(*t*) is related to $x^2(t)$, it was anticipated that the data would follow an approximately log-normal distribution. It transpired that, for the transformed data sets, and using the tests noted above, no data set deviated significantly from normality or sphericity.

Statistical analysis was carried out using SPSS software (IBM Corp., USA).

## Results

**Fig. 1:**
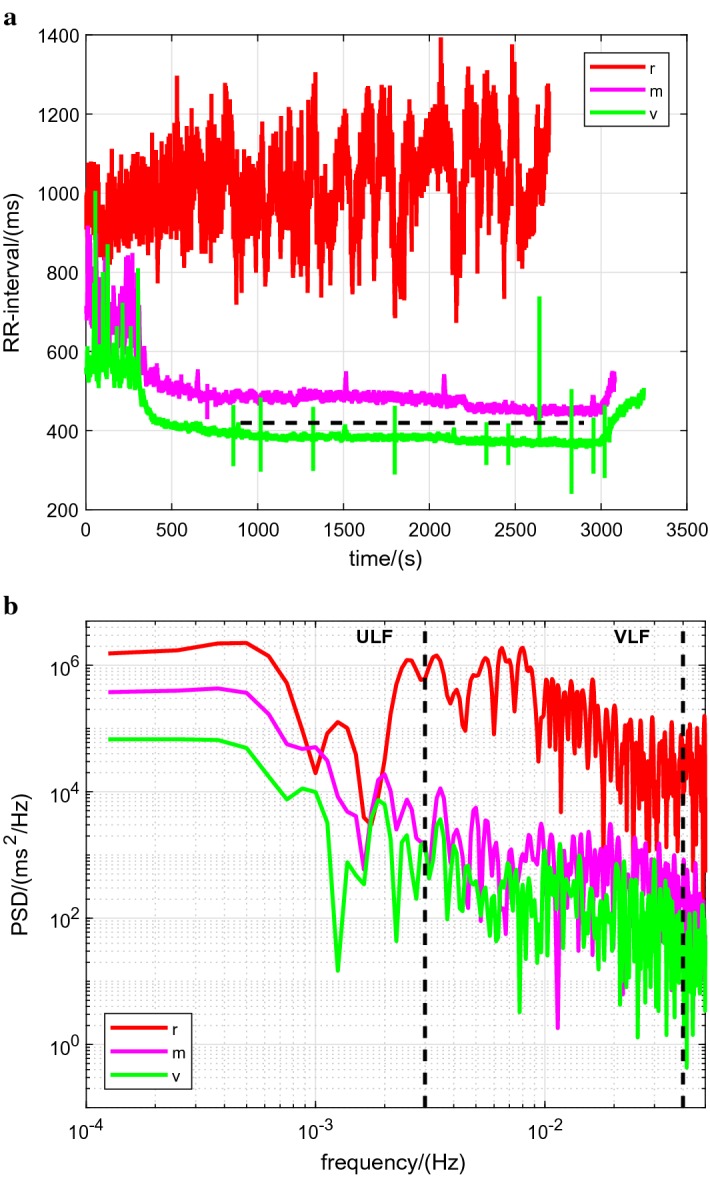
Data records for a single participant at the three intensity levels. *r* rest, *m* moderate, *v* vigorous. (**a**) Raw RR intervals. The horizontal dashed line depicts the border between moderate and vigorous intensity (60% of HRR) for this participant: HR = 143 bpm, RR interval = 420 ms. (**b**) Lomb-Scargle PSD estimates. The vertical dashed lines delineate the ultra-low frequency (ULF), very-low frequency (VLF) and low-frequency bands

**Fig. 2:**
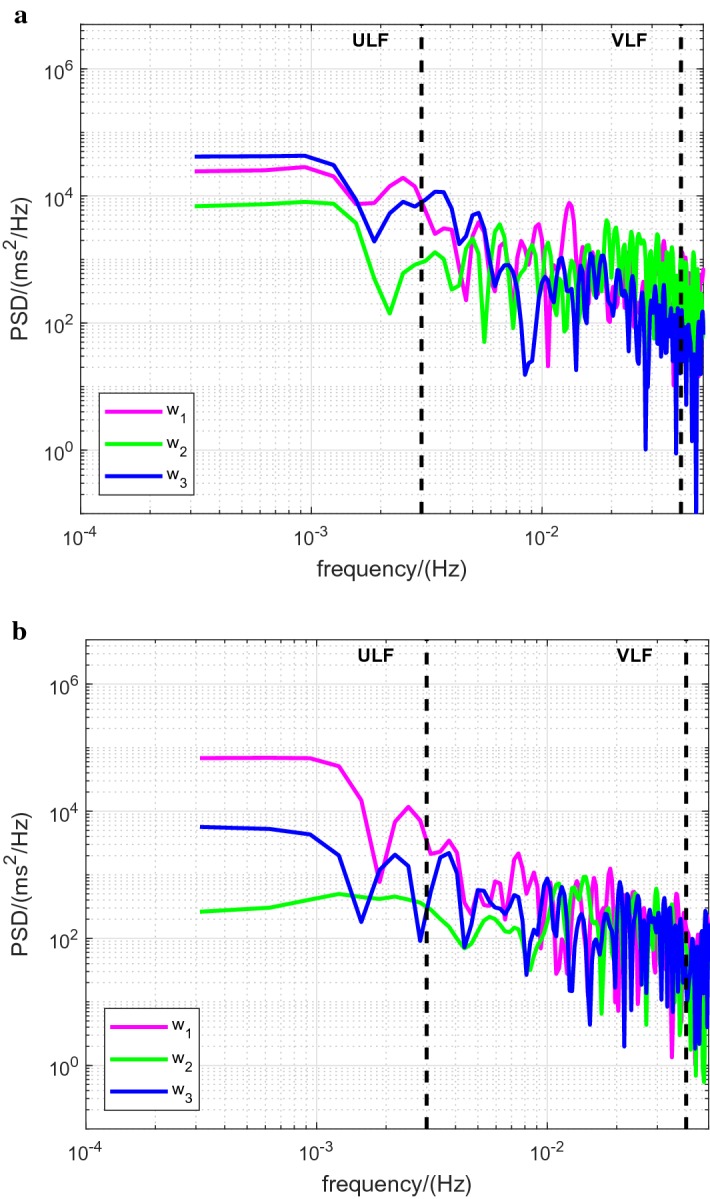
Lomb-Scargle PSD estimates for a single participant for the three time windows $w_1$, $w_2$ and $w_3$ at moderate (**a**) and vigorous (**b**) intensities. The vertical dashed lines delineate the ultra-low frequency (ULF), very-low frequency (VLF) and low-frequency bands

For illustration, data records for a single participant are provided (Figs. 1 and 2): these show the raw RR intervals recorded for the resting, moderate and vigorous intensity levels (Fig. 1a) and the corresponding Lomb-Scargle periodograms focused on the ULF and VLF bands (Fig. 1b); also shown are the power spectral density (PSD) estimates for this participant for the three time windows $w_1$, $w_2$ and $w_3$ at moderate (Fig. 2a) and vigorous (Fig. 2b) intensities.

### Dependence of HRV on intensity

**Fig. 3:**
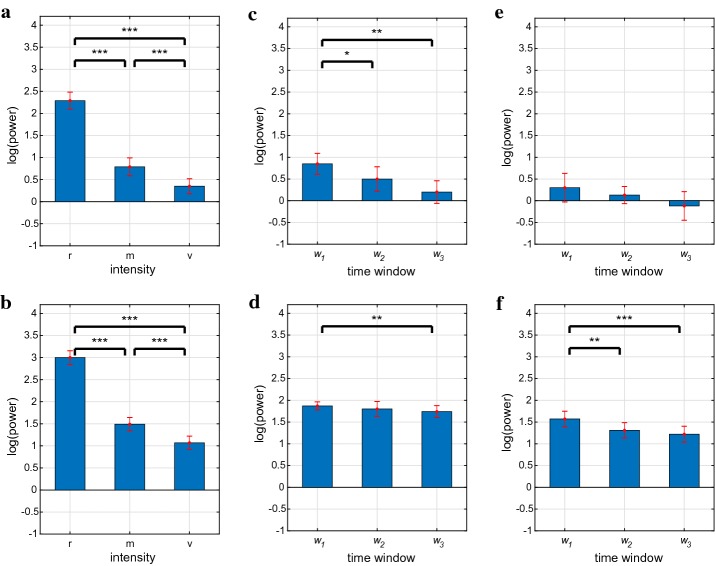
Mean power in the ULF (top row) and VLF (bottom row) frequency bands; the corresponding numerical values are given for intensity dependence (figure parts **a** and **b**) in Table 1 and for time dependence (figure parts **c**–**f**) in Table 2. The main bars depict mean power and error bars show 95% confidence intervals for the means. Values are given as $$\log _{10}([\text {power}/(\text {ms}^2)]/[1/(\text {ms}^2)])$$. $$^{*}\Leftrightarrow p < 0.05$$, $$^{**}\Leftrightarrow p < 0.01$$, $$^{***} \Leftrightarrow p < 0.001$$. *ULF* ultra-low frequency, *VLF* very-low frequency, *r* rest, *m* moderate, *v* vigorous. $$w_1$$, $$w_2$$ and $$w_3$$: first, second and third time windows.** a** Intensity dependence, ULF, **b** Intensity dependence, VLF, **c** Time dependence, m, ULF, **d** Time dependence, m, VLF, **e** Time dependence, v, ULF and **f** Time dependence, v, VLF

For both the ULF and VLF frequency bands, mean power was found to be significantly different between the resting, moderate and vigorous intensity levels (overall $p < 0.001$; Table 1). Paired comparisons showed that mean power was lower for the conditions moderate vs. rest ($p < 0.001$), for vigorous vs. rest ($p < 0.001$), and for vigorous vs. moderate ($p < 0.001$) (Table 1; Fig. 3a, b).

The reduction in power with increasing intensity is clearly reflected in the single-participant data records in both the time domain (lower dispersions in the respective RR intervals, Fig. 1a) and in the frequency domain (smaller areas under the PSD curves, Fig. 1b).

### Dependence of HRV on time, moderate intensity

For the moderate-intensity running condition, ULF mean power was found to be significantly different between the three time windows $w_1$, $w_2$ and $w_3$ (overall $p < 0.001$; Table 2). Similarly, at moderate intensity, VLF mean power was significantly different between the three time windows (overall $p = 0.041$; Table 2).

For ULF, paired comparisons showed that mean power was lower for the time windows $w_2$ vs. $w_1$ ($p = 0.031$) and for $w_3$ vs. $w_1$ ($p = 0.001$) (Table 2; Fig. 3c).

For VLF, paired comparisons showed that mean power was lower for the time window $w_3$ vs. $w_1$ ($p = 0.007$) (Table 2; Fig. 3d).

The observed differences can be discerned in the single-participant data record for moderate intensity (Fig. 2a, ULF and VLF bands).

### Dependence of HRV on time, vigorous intensity

For the vigorous-intensity running condition, ULF mean power did not differ significantly between the three time windows $w_1$, $w_2$ and $w_3$ (overall $p = 0.12$; Table 2; Fig. 3e).

For the VLF frequency band, and while running at vigorous intensity, mean power was found to be significantly different between the three time windows $w_1$, $w_2$ and $w_3$ (overall $p < 0.001$; Table 2). Paired comparisons then showed that mean power was lower for the time windows $w_2$ vs. $w_1$ ($p = 0.001$) and for $w_3$ vs. $w_1$ ($p < 0.001$) (Table 2; Fig. 3f).

For the VLF band, the observed differences between the time windows can be discerned in part in the single-participant data record for vigorous intensity (Fig. 2b, VLF band).

**Table 1:**
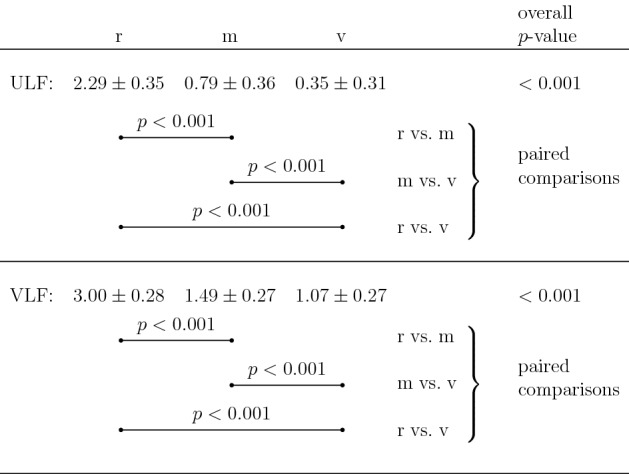
Power in the ULF and VLF frequency bands: intensity dependence (see also Fig. 3a, b)

$$n = 15$$

Values are mean ± standard deviation, given as $\log _{10}([\text {power}/(\text {ms}^2)]/[1/(\text {ms}^2)])$

*r* rest, *m* moderate, *v* vigorous, *ULF* ultra-low frequency, *VLF* very-low frequency

Paired comparisons: *p*-values adjusted using Bonferroni correction

**Table 2:** Power in the ULF and VLF frequency bands: time dependence at moderate and vigorous intensities (see also Fig. 3c–f)

	$w_1$	$w_2$	$w_3$	overall *p*-value	*p*-values, paired comparisons		
					$w_1$ vs. $w_2$	$w_1$ vs. $w_3$	$w_2$ vs. $w_3$
m							
ULF	$0.85 \pm 0.48$	$0.50 \pm 0.57$	$0.20 \pm 0.53$	< 0.001	0.031	0.001	0.255
VLF	$1.87 \pm 0.19$	$1.80 \pm 0.35$	$1.74 \pm 0.27$	0.041	0.778	0.007	0.704
v							
ULF	$0.30 \pm 0.59$	$0.13 \pm 0.36$	$-\,0.12 \pm 0.60$	0.12	−	−	−
VLF	$1.57 \pm 0.32$	$1.31 \pm 0.32$	$1.22 \pm 0.33$	< 0.001	0.001	$<0.001$	0.292

$$n = 18$$ (m); $$n = 15$$ (v)

Values are mean ± standard deviation, given as $\log _{10}([\text {power}/(\text {ms}^2)]/[1/(\text {ms}^2)])$

$w_1$ first window, $w_2$ second window, $w_3$ third window, *m* moderate, *v* vigorous, *ULF* ultra-low frequency, *VLF* very-low frequency

Paired comparisons: *p*-values adjusted using Bonferroni correction (presented only where overall *p*-value $< 0.05$)

## Discussion

The aim of this work was to investigate changes in ULF and VLF heart rate variability with respect to exercise intensity and time during treadmill running.

Substantial and significant decreases in both ULF and VLF power were observed as intensity increased from rest to moderate-intensity running and then to vigorous running. These findings of decreasing HRV are generally consistent with previous studies included in the review by Michael et al. [15], albeit those studies focused only on LF, HF and total power components. For example, the study of Tulpo et al. [19] reported a nonlinear decay of HRV as a function of exercise intensity: subjects had a decrease of LF and HF power from rest to light and moderate exercise intensity on a cycle ergometer. The study found a very small but significant decrease in LF power from moderate to vigorous exercise (80% of maximal oxygen uptake) and no significant decrease in LF and HF power thereafter. However, similar to most physiological studies, this article mainly focused on investigation of cardiac sympathetic and parasympathetic nervous system responses using relatively short-term recordings, so there was no report on ULF and VLF power.

Other studies have previously observed that VLF power increased during rhythmic activity (alternating rest and mild exercise) and normal/random activity when compared to rest [20], and that ULF power was higher during activity (ADL—activities of daily living) than at rest [21]. These apparent anomalies might be explained by the exercise conditions studied being of very low intensity when compared to the moderate running condition investigated in the present work: the intensity of the exercise conditions employed in those studies is not likely to have been substantially different from the resting intensity.

Significant decreases were observed over time in ULF and VLF power for the moderate-intensity running condition. At vigorous intensity, VLF power decreased significantly, while the observed decrease in ULF power was not significant. The latter might be explained by the fact that absolute ULF power levels at vigorous intensity were already very low during the first time window, thus making subsequent changes more difficult to detect. These observations are consistent with the results of previous studies which noted decreases over time in time-domain measures of HR-related signal intensity while using feedback control of HR during moderate-to-vigorous treadmill exercise of similar duration [13, 14].

Attenuation of HRV during exercise was also found in a previous study where subjects ran on a treadmill at an intensity of 60% of peak oxygen uptake: the LF and HF components were significantly higher at rest compared with exercise. Additionally, LF was significantly higher at the beginning of exercise (25–30 min of exercise) compared to near the end of exercise (85–90 min of exercise) [22].

It was suggested elsewhere that the mechanism for these time-related findings may not be the direct effect of exercise duration itself on HRV, but it may be due to dehydration, thus leading to reduced stroke volume and increased heart rate (cardiovascular drift) [15]. This concept is however challenged by the findings of Hunt et al. [13, 14], where heart rate was maintained constant over an exercise duration of 45 min by means of feedback control and automatic adaptation (reduction) in treadmill speed: despite the HR intensity staying constant, time-domain measures of HR tracking error and average control signal power were still seen to decrease. Since the latter studies used indirect time-domain measures of HRV, further investigations are recommended which combine feedback control of HR with direct frequency-domain analysis of RR intervals to more precisely elucidate the mechanisms underlying time-dependent changes in HRV.

The results of the present study have important implications for the design and analysis of feedback systems for automatic control of HR during treadmill exercise. HRV can be considered as a disturbance term that enters the feedback loop. This results in two main effects: HR will tend to deviate from the target HR level and this must be corrected by feedback action; and the HRV disturbance might, as a consequence of the feedback, cause unacceptable changes in the treadmill speed command [13]. The main challenge in feedback design is to achieve an acceptable tradeoff between accuracy of target HR tracking and control signal intensity.

The focus in the present work on ULF and VLF heart rate variability is important also for the design of exercise programmes and strategies. This is because these components of HRV dominate during long-duration exercise, and must be accounted for by appropriate design of the feedback control system for heart rate. LF and HF components, on the other hand, are less critical for two reasons: LF and HF power are lower than ULF and VLF power; disturbances within the LF and HF frequency bands will generally be attenuated in a natural way due to the low-pass characteristics of typical feedback designs.

The present work shows that it is most critical to meet these demands for low intensity exercise, and during the initial stages of any exercise bout, because the degree of HRV is higher under these conditions; for higher levels of exercise intensity, and/or as exercise progresses over time, reduction in HRV might make it possible to adaptively increase the feedback bandwidth to improve the accuracy of HR tracking performance but without necessarily provoking unacceptable levels of control signal activity.

## Conclusions

The degree of HRV in terms of ULF and VLF power was found to decrease with increasing intensity of exercise. HRV was also observed to decrease over time, but it remains to clarify whether these changes are due to time itself or to increases in HR related to cardiovascular drift.

With regard to design of feedback controllers for HR, these results suggest that attention should be focused on meeting performance targets at low intensity and during the early stages of exercise. Further studies of HR control are warranted in order to systematically investigate this feedback-design issue.

## Abbreviations

ANOVA: analysis of variance
bpm: beats per minute
ECG: electrocardiogram
HF: high frequency
HR: heart rate
HR_max_: maximal heart rate
HR_rest_: resting heart rate
HRR: heart rate reserve
HRV: heart rate variability
LF: low frequency
PSD: power spectral density
QRS complex: the combination of the Q, R and S wave deflections in the ECG
RMS: root mean square
RR interval: time between peaks in the QRS complex of the ECG wave
ULF: ultra-low frequency
VLF: very-low frequency
